# A Medical College Sued

**Published:** 1884-07

**Authors:** 


### A MEDICAL COLLEGE SUED.

Suit has been entered against the Georgia Eclectic Medical College of this city for $15,000.

It is alleged that the Faculty violated the State law by graduating students who had not attended two full courses of lectures.

